# AAV for gene therapy drives a nephrotoxic response via NFκB in kidney organoids

**DOI:** 10.1038/s41392-025-02336-2

**Published:** 2025-08-08

**Authors:** Navin Gupta, Ke Zhang, Venkata Sabbisetti, Jian Shu, Ryuji Morizane

**Affiliations:** 1 Nephrology Division, Department of Medicine, Mass General Brigham, Harvard Medical School, Boston, MA USA; 2 Cutaneous Biology Research Center, Massachusetts General Hospital, Harvard Medical School, Boston, MA USA

**Keywords:** Stem-cell differentiation, Gene therapy

**Dear Editor,**

Genome editing holds potential to cure human diseases. The first CRISPR/Cas9 gene therapy, Casgevy, may offer a definitive treatment for hemoglobinopathies through autologous transplantation of ex vivo genome-corrected hematopoietic stem cells. However, in vivo editing of solid tissues has been marred by organ failures and deaths, poorly predicted by animal studies.^1^ The lack of reliable preclinical tools for human-specific therapies, such as genome editing, has remained a critical unmet need. Preclinical safety testing of genome editing could benefit from complex tissue models that bear the human genome and reconstitute human responses. Given the kidney’s susceptibility to toxic reactions from investigational drugs, studies using human stem cell-derived kidney organoids may inform clinical trials and improve patient outcome.^2^ Here, kidney organoids serve as a preclinical testing platform for CRISPR/Cas9 genome editing via adeno-associated virus (AAV) delivery, a common strategy in clinical trials that have raised safety concerns due to viral toxicity.^1^

Gene therapy requires a delivery system for the specific and efficient transport of the payload to its target. Viral delivery using AAVs has been a leading delivery system, owing to a variety of serotypes that specify tissue tropism, its minimal pathogenicity, and its capability for long-term gene expression. Numerous AAV serotypes have been leveraged by FDA-approved gene therapies despite the consistently high seroprevalence of pre-existing AAV neutralizing antibodies, against a variety of serotypes and across diverse ethnic backgrounds.^3^ To assess the pharmacodynamics of AAV contacting nephron epithelia, kidney organoids were bathed in AAV serotypes of reported tropism for the mammalian kidney from differentiation day 21. Experimental duration and AAV dosage (multiplicities of infection; MOI) were determined by longitudinal live epifluorescent microscopy. AAV2 outperformed the negligible organoid tropism seen with AAV8 and AAV9, delivering *GFP* transgene to 46.2 ± 4.7% of *LTL*^*+*^ proximal tubules, 45.8 ± 12.5% of *CDH1*^*+*^ distal tubules, 14.6 ± 1.6% of *PDGFR-β*^*+*^ mesenchymal cells, 3.7 ± 1.7% of *CD31*^*+*^ endothelial cells, and 1.8 ± 0.4% of *PODXL*^*+*^ podocytes (Fig. 1a).

**Fig. 1:**
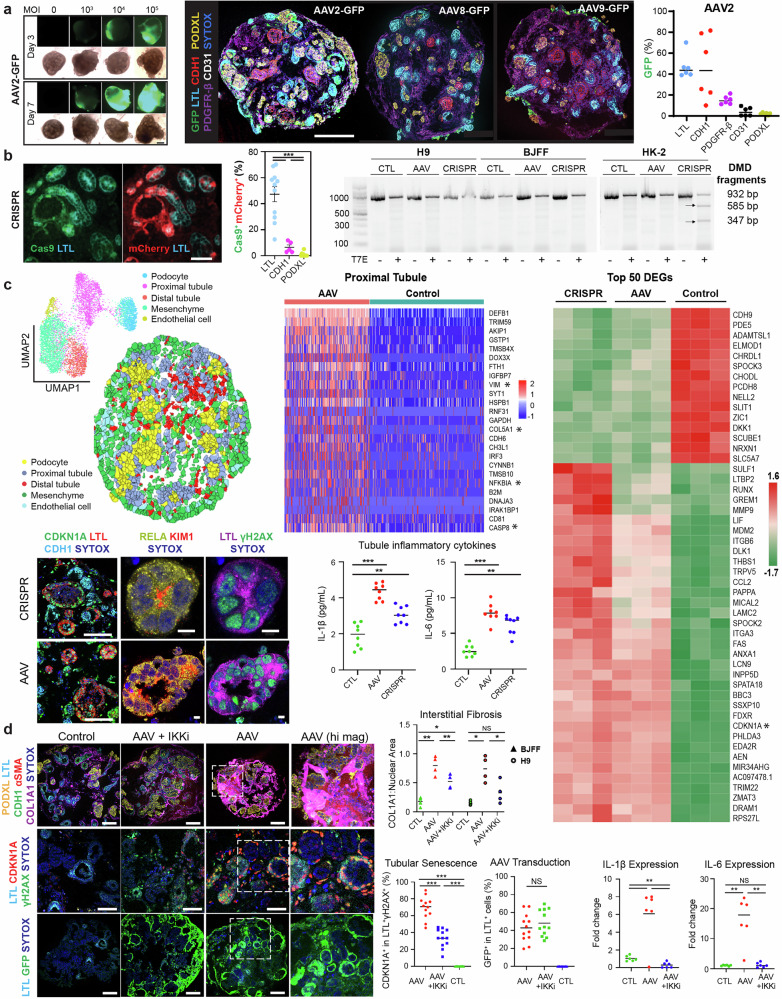
AAV administration drives a nephrotoxic response via NFκB in kidney organoids. **a** *Left*: Live epifluorescent microscopy informing AAV dose and duration (AAV2 depicted). *Right*: Multiplexed immunostaining and quantification of *GFP* reporter delivery in *LTL*^*+*^ proximal tubules, *CDH1*^*+*^ distal tubules, *PDGFR-β*^*+*^ interstitial cells, *CD31*^*+*^ endothelia, and *PODXL*^*+*^ podocytes using variable of AAV serotypes. Scale bar 200 μm. **b** *Left*: Immunostaining and quantification of AAV2-based delivery of Cas9 and mCherry in the nephron epithelial cells of CRISPR samples. Scale bar 50 μm. ***p < 0.001. *Right*: Gel electrophoresis of products from the T7 endonuclease performed on CRISPR, AAV, and control samples in kidney organoids from both H9 female embryonic stem cells and BJFF male-induced pluripotent stem cells, as well as HK-2 human immortalized proximal tubules. **c** *Top left*: UMAP representation of clusters of all samples including CRISPR, AAV empty vector, and control and spatial visualization of cell clusters of the AAV empty vector sample based on STARmap probes that included marker genes to resolve five distinct cellular clusters. *Top middle*: Heatmap of the top 25 upregulated DEGs in proximal tubules by spatial transcriptomics between AAV empty vector and control samples. *Right*: Heatmap of the top 50 DEGs by bulk RNAseq between CRISPR, AAV, and control in biologic triplicates. *Bottom*: Immunostaining of CDKN1A, RELA, γH2AX, and KIM1 in proximal tubules of AAV and CRISPR organoid samples and Mesoscale detection of *IL-1β* and *IL-6* in organoid supernatants of control, AAV, and CRISPR samples. Scale bars 100 and 5 μm. ***P < 0.001. **d** *Left*: Immunostaining of *COL1A1* interstitial deposition (*top*, scale bar 100 μm), *CDKN1A* positivity in injured proximal tubules (*middle*, scale bar 50 μm), and *GFP* reporter delivery (*bottom*, scale bar 100 μm) in AAV-treated samples with and without IKK inhibition, as compared to controls. *Right*: Quantification of *Left* and RT-qPCR of *IL-1β* and *IL-6* in similarly treated samples. ***p < 0.001, **p < 0.01, NS not significant

AAV2 was chosen to deliver *staphylococcus aureus* Cas9 (saCas9) and guide RNA (gRNA) to kidney organoids, via a commonly used dual vector strategy that overcomes size limitations of AAV-based delivery.^4^ The *DMD* locus was targeted with two gRNAs to coordinate a deletion spanning introns 44 to 55 that restores the reading frame, a theoretical treatment for 65% of Duchenne’s Muscular Dystrophy cases. Fluorescent-labeling of the gRNA vector with mCherry, combined with saCas9 immunostaining, reflected co-delivery to 47.2 ± 5.7% of *LTL*^*+*^ proximal tubules as compared to modest amounts of *CDH1*^+^ and *PODXL*^+^ cells. Given the co-delivery of saCas9 and gRNA to nearly half of the proximal tubules in CRISPR samples, a T7 endonuclease assay was used to detect indel-related DNA mismatches in kidney organoids from both female embryonic stem cells (H9) and male-induced pluripotent stem cells (BJFF). This assay failed to detect on-target DNA mismatches, possibly related to the inaccessibility of the *DMD* locus, while HK-2 immortalized proximal tubules demonstrated ~50% on-target editing (Fig. 1b). As the T7 endonuclease assay was designed to detect indels in genomic DNA, but not our desired *DMD* deletion, RNA analysis via spatial transcriptomics (ST) was conducted with specific pobes including *DMD*. Additionally, CRISPR samples were standardized to empty-vector AAV, and empty-vector AAV was standardized to control, for safety profiling of genome editing and AAV transduction, respectively.

STARmap (spatially-resolved transcript amplicon readout mapping), an ST method coupling hydrogel technology with mRNA rolling amplification and in situ sequencing and in situ hybridization,^5^ included a probe for wild-type *DMD* designed to target the junction of exons 44 and 45. Although wild-type *DMD* was poorly expressed at 0.02%-0.03% across cell types, a modest tubular reduction in CRISPR compared to empty-vector AAV samples suggested a quite limited and non-significant degree of on-target editing. Regarding safety profiling, 1486 customized STARmap probes targeting 496 genes included differentially expressed genes (DEGs) in injured proximal tubules and mesenchymal cells in organoids (Zenodo: 10.5281/zenodo.15742418). STARmap resolved a similar profibrotic phenotype in proximal tubules of both CRISPR and empty-vector AAV samples, with DEGs by Model-based Analysis of Single-cell Transcriptomics indicating that AAV alone drove partial epithelial-to-mesenchymal transition (*VIM), interstitial fibrosis (*COL5A1), apoptosis (*CASP8), and NF-κB-related inflammation (*NFKBIA), as standardized to vehicle control. Similarities in the gene expression profiles of CRISPR “loaded” compared to empty-vector AAV “unloaded” samples confirm that AAV infection itself, as opposed to genome editing, drives transcriptional differences between samples. A profibrotic and senescent (*CDKN1A) phenotype was common to “loaded” and “unloaded” samples by whole RNA sequencing (GEO GSE286037). Next, immunostaining confirmed *γH2AX* and *CDKN1A* positivity localized to *LTL*^*+*^ proximal tubules, which manifest *RELA* of canonical NF-κB signaling as a potential druggable therapeutic pathway. The extensive DNA damage in these highly transduced tubules likely stems from the DNA release step of AAV infection, where viral uncoating triggers nuclear stress, genotoxicity, and a strong DNA damage response. IL-1β and IL-6, biomarkers of tubular injury and activators of the NF-κB cascade, were also detected in organoid supernatants to establish AAV transduction as a primary instigator of a nephrotoxic response (Fig. 1c). As the genomic differences amongst AAV serotypes are often limited to the tropism-defining variable region of the capsid sequence, namely viral protein 3 (*VP3*), the nephrotoxic results of AAV2 may extend beyond AAV2 to any naturally-occurring or synthetic serotype with tubular tropism.

Mechanistic testing was conducted using bardoxolone, an FDA safety-approved agent and potent inhibitor of the IKK kinase complex central to canonical NF-κB signaling. Following concurrent IKK inhibition (IKKi) during AAV transduction (day 21 to 28), longitudinal monitoring to day 42 found that IKKi significantly rescued AAV-induced COL1A1^+^ interstitial fibrosis in kidney organoids from both male and female, embryonic and induced pluripotent stem cells. Reduced fibrosis correlated with IL-1β and IL-6 expression falling to control levels, whereas senescence in injured tubules reduced from 69.2 ± 6.6% to 33.4 ± 5.5%. Although continued treatment to day 42 may have been more beneficial, IKKi did not mediate its significant nephroprotective effect by limiting AAV transduction, which would be counterintuitive to genome editing, as evidenced by the preserved transgenic delivery of *GFP* to tubules (Fig. 1d).

The translational utility of kidney organoids may be limited by their immaturity, which may over- or under-estimate the tropism and toxicity of AAVs in the kidney. Additionally, the lack of physiologic vasculature in organoids, which prevents the assessment of different AAV administration routes (e.g., intravenous, intra-arterial, retrograde ureteral), and the inability to mimic glomerular filtration dynamics, poses further challenges. Although AAVs are below the size threshold of glomerular sieving at ~25 nm, systemically delivered viral particles would largely traverse the glomerulus and reach the peritubular capillary bed since the glomerular filtration fraction is typically 20%. Here kidney organoids were bathed in viral particles to bring AAV in contact with the basolateral membrane of tubules, a region typically supplied by the peritubular capillary bed. Despite these limitations, the model still offers valuable insights into AAV tropism and toxicity, especially regarding proximal tubule effects. Future improvements, such as incorporating vascularized structures, may further enhance the physiological relevance of these models.

Genome editing holds great promise to revolutionize medicine through curative treatment, however, early efforts have raised unanticipated safety concerns in clinical trials. A review of 255 AAV-based gene therapy trials reported 11 patient deaths, independent of the AAV serotype used or route of administration.^1^ Clinical translation of genome editing may benefit from therapeutic strategies delineated in preclinical studies. Using kidney organoids, we find that AAV transduction induces genotoxicity and IL-1β secretion that activates the canonical NFκB pathway, whose inhibition halts interstitial fibrosis by preventing tubular senescence. When targeting alternative organs, IKKi may prevent AAV nephrotoxicity due to off-target kidney tropism. Beyond safety testing, kidney organoids may be an optimal preclinical platform for on-target efficacy testing, as tubulopathies largely comprise the >600 monogenic kidney diseases which constitute 50% of CKD in children and 30% of non-diabetic CKD in adults. Our findings demonstrate the utility of kidney organoids as a preclinical testing platform for genome editing, which may inform clinical trials of strategies that limit organ failures and death.

## Supplementary information

Supplementary Material

## Data Availability

Spatial transcriptomic data were deposited into Zenodo under accession number 10.5281/zenodo.15742418. Whole transcriptomic data was similarly deposited under accession number GSE286037. All data supporting the findings of this study are available in the main text and its supplementary information.
