# Supplementary material for: AAV for gene therapy drives a nephrotoxic response via NFκB in kidney organoids
Source: Signal Transduct Target Ther. 2025 Aug 8;10:252. doi: 10.1038/s41392-025-02336-2 (PMC12332185; doi:10.1038/s41392-025-02336-2)
Supplement: Supplementary file 1 — Supplementary Material [file 41392_2025_2336_MOESM1_ESM.docx]

Supplementary Materials for

AAV for gene therapy drives a nephrotoxic response via NFκB in kidney organoids

Navin Gupta, Ke Zhang, Venkata Sabbisetti, Jian Shu, Ryuji Morizane

Correspondence to: rmorizane@mgh.harvard.edu

**This PDF file includes:**

Materials and Methods

**Materials and Methods**

**Nephron organoid generation.** Human pluripotent stem cell culture, directed differentiation to kidney organoids, and kidney organoid maintenance was performed as previously reported, noting each organoid was generated in suspension culture by transferring 10^5^ adherent cells on differentiation day 8 [2].

**AAV infection:** AAV2-GFP, AAV8-GFP, and AAV9-GFP were obtained from the University of Iowa viral vector core and kidney organoids were treated at multiplicities of infection (MOI) of 10^5^ viral genomes (vg)/cell from differentiation day 21 - 28 (**Figure 1a**). Kidney organoids were similarly treated with custom VectorBuilder AAV2 vectors which separately delivered Cas9 (AAV2-CMV-saCas9; VB180125-1089ffa) and gRNA (AAV2-U6-gRNA-CMV-mCherry; VB210506-1386zxe). Samples denoted ‘CRISPR’ involve co-infection of these vectors at cumulative MOI 10^5^ vg/cell and acute effects evaluated on differentiation day 28 (**Figure 1b,c**). Following AAV2-empty vector + IKKi (day 21 – 28), longitudinal testing was conducted in vehicle media until differentiation day 42 (**Fig. 1d**).

**Immunostaining of kidney organoids**. Wholemount and frozen section immunostaining was performed as previously reported [2]. Antibodies used were for *α-SMA* (Sigma; F3777; DF 1:500), *CD31* (Abcam, ab9498, DF 1:200), *CDH1* (Abcam, ab11512, DF 1:500), *CDKN1A* (Sigma, sab5700742, DF 1:200), *COL1A1* (Rockland, 600-401-103-0 1, DF 1:200), *GFP* (Sigma, ab13970, DF 1:200), *KIM1* (R&D systems, AF1750, DF 1:200), *LTL* (Vector labs, B-1325, DF 1:200), *mCherry* (Sigma, ab167453, DF 1:200), *MEIS1/2/3* (Active motif, 39796, DF 1:100), *PDGFR-β* (R&D systems, AF385, DF 1:200), *PODXL* (R&D systems, AF1658, DF 1:500), *RELA* (Rockland, 100-401-266, DF 1:200), *saCas9* (ThermoFisher, A01951-40, DF 1:200), *SIX2* (Proteintech, 11562-1-AP, DF 1:500), *SYTOX blue* (Invitrogen, S11348, DF 1:500), *γH2AX* (Cell signaling, 2577, DF 1:100).

**RT-qPCR of whole organoid lysates.** RNA isolation, cDNA library preparation, primer design, RT-qPCR performed, and data normalization and visualization was performed in biologic replicates as previously reported [2]. RT-qPCR was performed on *IL-1β* (Forward: 5’-ATGATGGCTTATTACAGTGGCA-3’; Reverse: 5’-GTCGGAGATTCGTAGCTGGA-3’), *IL-6* (Forward: 5’-CCTGAACCTTCCAAAGATGGC-3’; Reverse: 5’- TTCACCAGGCAAGTCTCCTCA-3’), *GAPDH* (Forward: 5’- CAATGACCCCTTCATTGACC-3’; Reverse: 5’-GACAAGCTTCCCGTTCTCAG-3’). Genomic DNA PCR was formed on *DMD* (Forwards: 5’-CCACCAAAACAGTGACAATCCA-3’; Reverse: 5’-AGGGTCTCAGGCTTGTATGTC-3’).

**Statistical analysis**. Dot plots are generated by the standard convention of each dot representing a biologic replicate and the center line indicating the mean. To limit the effects of intra-batch organoid heterogeneity, multiple biologic replicate organoids (n = 3-6) from a given batch were used across all test and control conditions for each experiment. To limit the effects of inter-batch organoid heterogeneity, quintuplicate experiments were performed on independent batches of organoids to encompass the results of transgenic GFP delivery, Cas9 and gRNA delivery, AAV-empty vector vs CRISPR experiments, spatial transcriptomics, and IKK inhibition studies. To limit the differential effects between stem cell lines and male/female gender, we duplicated experiments using kidney organoids derived from male iPSCs (BJFF.6) and female ESCs (H9) for transgenic GFP delivery, Cas9 and gRNA delivery, and IKK inhibition studies to yield similar results. Quantification of Cas9 and mCherry was performed by unbiased gating for nephron epithelial (LTL, CDH1, PODXL) and then adding the Cas9 and mCherry fluorophores in a blinded fashion. Quantification was performed using ImageJ for areas of COL1A1, standardized to the DAPI area**.** Statistical analysis was performed in Excel with statistical significance by two-tailed Tukey’s pairwise comparison tests. Different significance levels (p values) are indicated with asterisks by the convention, * represents < 0.05, ** represents < 0.01, and *** represents < 0.001. GraphPad Prism was used for visualization.

**T7 Endonuclease assay.** Genomic DNA was extracted from kidney organoids and HK-2 immortalized human proximal tubules using microLYSIS-PLUS (Cat. No. 2MLP-50, Cambio), per the manufacturer’s protocol. Genomic DNA PCR amplified a 932 base pair amplicon of *DMD* flanking gRNA target site by 585 and 347 base pairs. Amplicons were loaded on a gel and the corresponding band was digested, DNA eluted (Qiagen gel extraction kit), and subject to T7 endonuclease I digestion. Samples were then loaded on a 1.5% agarose gel and gel electrophoresis performed.

**Spatial transcriptomic probe design.** SNAIL probes were designed as follows: (1) For genes with multiple transcript isoforms, only the shortest isoforms were considered and the coding regions were used except for non-coding RNAs; (2) Picky 2.2 was used to design the hybridization sequence of each probe pair with length restriction of 40-46 nucleotide; 3 sequences for each gene were designed; (3) the resulting complementary DNA (cDNA) sequences (40-46 nt) were split into halves of 20-25 nt, with an 0-2 nt gap in between, and with the best match of melting temperature (Tm) between the two halves. Homemade sequencing reagents included six reading probes (R1 to R6) and fluorescent probes labeled with Alexa 488, 546, 594 and 647. All the probes were under 60 nt and manufactured as by Integrated DNA Technologies (IDT).

**STARmap sample preparation.** Fresh kidney organoids were immediately embedded in O.C.T. and snap-frozen. Tissues were either stored at -80° C or transferred to the cryostat and cut as 10-µm cryosections.

**STARmap.** STARmap was performed and data was processed based on methods illustrated in [5]. Images were acquired by Leica Stellaris 5 confocal microscope with a 405 diode, white light laser and ×40 oil-immersed objective (numerical aperture 1.3).

**STARmap single-cell data analysis.** The resulting count matrices were analyzed using Seurat (v4.3.0) in R (v4.2.2). For each sample, cells with fewer than 30 transcripts or 20 genes were filtered out. The remaining cells were normalized using the SCTransform function (with vst.flavor = "v2" among other default parameters), followed by Principal Component Analysis (PCA). Subsequently, the datasets were integrated. The functions SelectIntegrationFeatures, PrepSCTIntegration, and FindIntegrationAnchors (with normalization.method = "SCT" among other default parameters) were used. Finally, the datasets were integrated using the IntegrateData function (with normalization.method = "SCT"). Default parameters were used for the FindNeighbors, FindClusters, and RunUMAP functions. The PrepSCTFindMarkers was used before to run the FindAllMarkers function using as assay “SCT”. Cluster annotation was done using the Kidney Tissue Atlas data of Kidney Precision Medicine Project (<https://atlas.kpmp.org/>) as reference.

**Differential expression analysis between conditions.** Differential expression analysis between conditions was run on the integrated Seurat object using the “RNA” assay. The data were normalized using the NormalizeData function and the function FindMarkers was used with assay = "RNA", test.use ="MAST", min.pct = 0.01, min.cells.group = 10 parameters. Only Differential Expressed Genes (DEGs) with FDR < 0.05 and |avg_log2FC| > 1 were considered significant.

**Biomarker assays.**

The MSD protocol in U-Plex plates first involved the preparation of individual U-Plex Linker-Coupled antibody solutions. An individual biotinylated antibody was coupled to a unique linker, and the antibody identity was recorded next to the linker number on the spot map. Two hundred μl of each biotinylated antibody was added to 300 μl of the assigned linker. Samples were mixed by vortexing 3 times and incubated at room temperature for 30 minutes. Then two hundred μl of stop solution was added. Samples were mixed by vortexing 3 times and incubated at room temperature for 30 minutes. The multiplex coating solution was prepared with six hundred μl of each U-Plex linker-coupled antibody solution combined into a single tube and mixed by vortexing. Antibodies were pooled and brought to 600 μl by mixing with the stop solution to produce a final 1X concentration and mixed by vortexing. The U-Plex plate was then coated by adding fifty μl of 1X multiplex coating solution to each well. The plate was sealed with adhesive and placed in a shaker for 1 hour at room temperature at 900 rpm/min. The plate was washed 3 times using PBST with 300 μl/well 3 times for 5 mins each. Biomarker assays were then performed by adding fifty μl of prepared calibrator (with diluent 43) to diluted controls and neat samples in each well. Samples were gently tapped on all sides. The plate was sealed with adhesive and incubated at room temperature with shaking at 900 rpm/min for 1 hour. The plate was then washed 3 times with at least 300 μl/well of PBST for 5 mins each. Fifty μl of detection antibody solution was next added to each well. The plate was sealed with adhesive and incubated at room temperature with shaking at 900 rpm/min for 1 hour. Then the plate was washed 3 times with at least 300 μl/well of PBST for 5 mins each. Lastly, 150 μl of MSD Gold Read Buffer B was added to each well and the plate was analyzed on an MSD Quickplex SQ120.
